# Conventional Cells—The Last Step Toward General Acceptance of Standard Conventional Cells for the Reporting of Crystallographic Data

**DOI:** 10.6028/jres.107.030

**Published:** 2002-08-01

**Authors:** Alan D. Mighell

**Affiliations:** National Institute of Standards and Technology, Gaithersburg, MD 20899-8520

**Keywords:** *C*-centered cell, centered monoclinic cells, conventional cells, *I*-centered cell, mathematical lattices

## Abstract

In 1969, a seminal section on reduced forms and conventional cells was published in the *International Tables for X-Ray Crystallography*. The section contains a table that gives a metric classification of the 44 reduced forms. In 2001, this table with appropriate revisions was republished in the Journal of Research of the National Institute of Standards and Technology. An especially valuable feature of the table is that it defines and allows the user to determine a standard conventional cell. Since 1969, there has been an evolution toward acceptance and widespread use of such conventional cells. An inspection of the articles in key crystallographic journals reveals that most cells follow the conventions. However, one major exception remains—the centered monoclinic lattices. In approximately one-third of these cases, non-conventional *C*-centered cells are used, apparently to avoid the use of *I*-centered cells. It is recommended that the crystallographic community routinely use the *I*-centered conventional cell in such cases.

## 1. Introduction

In 1969, a seminal section [1] on reduced forms and conventional cells was published in the *International Tables for X-Ray Crystallography*. The section contains a table that gives a metric classification of the 44 reduced forms. In 2001, this table with appropriate revisions was republished in the Journal of Research of the National Institute of Standards and Technology [2]. An especially valuable feature of the table is that it allows the user to determine a standard conventional cell. From the nature of the reduced form, one can determine the reduced form number, Bravais lattice, and the transformation matrix to a *standard conventional* cell (or simply *conventional* cell). The table is widely used by the scientific community as it is imbedded in scientific software associated with automated x-ray diffractometers as well as in other distributed software.

This conventional cell is rigorously defined mathematically, unique, and can be calculated using a computer program. The cell is logical. For example, for those cases in which cell edges are not determined by symmetry, one selects the *shortest possible vectors*. Thus in the triclinic system, the reduced cell which is based on the shortest three non-coplanar vectors of the lattice is chosen with the edges obeying *a* ≤ *b* ≤ *c*. In the monoclinic system, the cell is based on the shortest possible vectors in the *ac* plane with *b* the unique axis. The angle *β* is taken as non-acute. This choice allows a primitive, a side-centered, and a body-centered lattice. In the primitive and body-centered lattices, *a* and *c* obey *a* < *c*. The side-centered lattice is taken as *C*-centered.

There are many advantages to using conventional cells for the reporting of crystallographic data. For example, one can readily compare cells to ascertain if a new compound is the same as or related to a previously reported material. Accordingly, to facilitate the evaluation and use of data, several of the crystallographic data centers transform each original literature cell to the *Crystal Data* (*CD*) cell (*CD* cell = the conventional cell or a closely related cell determined by relabeling of axes). For this purpose, the NBS*AIDS83 [3] program has been used for over 20 years. This program determines the *CD* cell directly from the literature cell. In a verification sequence, the program also calculates the *CD* cell from the reduced form using the table in the above references [1,2]. As NIST*LATTICE [4] is a powerful tool for the evaluation and determination of metric lattice symmetry via matrix methods [5], it is often used in the determination of symmetry and the reduced form especially for difficult cases.

A recommended goal for the crystallographic community would be to strive for the universal acceptance of standard conventional cells and to publish crystallographic results therein. This is especially important today, as so many cells are determined in so many laboratories and reported in such diverse journals. Use of such cells aids both the experimentalist, the journal reader, and the data centers as it eliminates the need to transform cells and coordinates in order to compare structural parameters. Fortunately, the crystallographic community is very close to achieving such a goal. As shown in the next section, proper determination of conventional cells to characterize a subset of the centered monoclinic lattices represents the final major hurdle.

## 2. Discussion

### 2.1 Conventional Cells: Current Status

Since 1969, there has been an evolution toward the general use of the standard conventional cells. An inspection of articles in key crystallographic journals (e.g. Acta Crystallographic Section C—Crystal Structure Communications) reveals that most cells reported in the literature follow the conventions^1^ specified in the above two articles [1,2]. For example, for the most populous space group (No. 14), the shortest vectors in the *ac* plane, with the *b*-axis unique, are usually selected which results in either the space group setting *P*2_1_/*c* or *P*2_1_/*n*. For triclinic crystals, the reduced cell is routinely used.

Both the editors of the *International Tables for Crystallography* and the instrument manufacturers have played an essential role in this evolutionary progress. Recent versions of Volume A of the *International Tables for Crystallography* [6] explicitly give the required space group settings to assist the experimentalist in the low symmetry systems. For example, the equivalent positions for *P*2_1_/*n* and *I*2/*a* are explicitly given. In addition, the software provided with many of the automated x-ray diffractometers makes it possible to readily obtain the conventional cell in the low symmetry crystal systems *for most cases*.

### 2.2 Conventional Cells: The Problem with Centered Monoclinic Cells

The general use of non-conventional *C*-centered monoclinic cells—when conventional *I*-centered cells should have been selected—remains the principal remaining problem. An understanding of the problem can be gained by inspection of Tables 1 and 2. Table 1 is derived from the table of 44 reduced forms published originally in the *International Tables for X-Ray Crystallography* [1] and later, with revisions, in the Journal of Research of NIST [2]. It contains the 13 reduced forms that correspond to centered monoclinic lattices. As the Table 1 shows, there are two possible standard conventional settings for the centered monoclinic cells—*C* and *I*. An analysis of approximately 11 000 centered monoclinic cells in the Crystal Data File [7] shows that the centered monoclinic literature cells transform to *I*-centered and *C*-centered conventional cells in one-third and two-thirds of the cases, respectively. However, an analysis of the authors’ cells reported in the literature shows that the *I*-centered cell is rarely used.

Today, this avoidance continues unabated as can be seen by inspection of recent publications in the major crystallographic journals. A review of all 80 centered monoclinic cells published in 2001 in Acta Crystallographic Section C, shows that the *I*-centered cell is still infrequently used to report the experimental results. A detailed analysis of recent publications shows that the conventions are not entirely ignored. In fact for most cases in which a *C*-centered cell is the conventional cell, it is indeed selected (i.e., in approximately two-thirds of the cases). But for those cases, in which the *I*-centered cell is the conventional cell, the author usually selects a non-conventional *C*-centered unit cell. Clearly there is an aversion to using the *I*-cell for those cases in which it is the conventional cell based on the shortest vectors in the *ac* plane (*b*-unique)!

Table 2 shows examples of the selection of the nonstandard *C*-centered cells by experimentalists [8–12]. Note that in each case, the *C*-centered cell can be transformed to an *I*-centered cell with a *β* angle closer to 90°. (To facilitate comparisons, the rows with *β* angles have been shaded.) In case 3, for example, the *C*-centered cell with a *β* angle of 131.61° can be transformed to an *I*-cell with a *β* of 91.41°. The selection of such highly skewed cells, such as the *C*-centered cell in case 3, is not recommended because it is often associated with experimental errors such as missed symmetry. The reluctance to use the *I*-centered cell is a bit puzzling in view of the fact that recent editions of Volume A of the *International Tables for Crystallography* explicitly give all the symmetry related positions for the *I*-cell. The most likely explanation is that the software associated with diffractometers continues to steer the experimentalist toward the *C*-centered cell even in those cases when it should be *I*.

## 3. Conclusion and Recommendation

Because many compounds crystallize in centered monoclinic lattices, the widespread use of the non-standard cells to characterize approximately one-third of these lattices is a nontrivial problem. An analysis of the NIST Crystal Data [7] shows that approximately 9 % of all compounds crystallize in lattices characterized by a centered monoclinic unit cell. In fact, space group 15 (*C*2/*c*, *I*2/*a*) is one of the more populous space groups.

Both *I*- and *C*-centered conventional unit cells can occur with monoclinic centered lattices. *The maximum in utility of crystallographic data will be achieved if, in each case, the experimentalist follows the conventions in Table [1,2] and selects the type of cell centering (I or C) that leads to a conventional cell based on the shortest vectors in the ac plane (b-axis unique)*. By discontinuing the use of a non-conventional *C*-centered cell with a *β* angle greater than that in the conventional *I*-centered cell, uniformity in crystallographic practice will be achieved. In all cases then, the practice for the monoclinic system would be to select a conventional cell based on the shortest vectors in the *ac* plane (*b*-axis unique) for primitive as well as centered cells.

An effective way to promote this harmonization of crystallographic data could be achieved if journals were to modify their rules and policies to require or, at least, recommend that the standard conventional cell be used for reporting the monoclinic centered lattices. Likewise journal editors and reviewers should support this policy in the review process. In addition, the diffractometer makers should modify and enhance their software so that experimentalists routinely are guided to select the *I*-centered cell when it is the standard conventional cell. Finally, other widely disseminated software packages, such as indexing programs, should be appropriately modified.

## Figures and Tables

**Table 1:** Metric classification of the 13 reduced forms^a^ corresponding to the monoclinic centered lattices. From the nature of the reduced form, one can determine the reduced form number and the transformation matrix to the conventional cell. To assure that one obtains a conventional cell^h^ based on the shortest translations in the *ac* plane, the conditions in the footnote for the specified centered monoclinic lattices must be checked. In those cases for which the transformation matrix in the footnote premultiples a given table matrix, the resultant cell centering is indicated in parentheses following the transformation matrix

Reduced form No.	Reduced form matrix				Reduced form type	Bravais lattice		Cell transformation reduced → conventional
	First row	Second row						
	*a*·*a b*·*b c*·*c*	*b*·*c*	*a*·*c*	*a*·*b*				
*a* = *b*								
10	***a*·*a a*·*a c*·*c***	***b*·*c***	***b*·*c***	***a*·*b***	+	Monoclinic	*C*^d^	$110/1\bar{1}0/00\bar{1}$
14	***a*·*a a*·*a c*·*c***	−|***b*·*c***|	−|***b*·*c***|	−|***a*·*b***|	−	Monoclinic	*C*^d^	$110/\bar{1}10/001$
17	***a*·*a a*·*a c*·*c***	−|***b*·*c***|	−|***a*·*c***|	−(***a*·*a***−|***b*·*c***|−|***a*·*c***|)	−	Monoclinic	*I*^e^	$\bar{1}0\bar{1}/\bar{1}\bar{1}0/011$
*b* = *c*								
20	***a*·*a b*·*b b*·*b***	***b*·*c***	***a*·*c***	***a*·*c***	+	Monoclinic	*C*^b^	$011/01\bar{1}/\bar{1}00$
25	***a*·*a b*·*b b*·*b***	−|***b*·*c***|	−|***a*·*c***|	−|***a*·*c***|	−	Monoclinic	*C*^b^	$011/0\bar{1}1/100$
*a* ≤ *b* ≤ *c*^g^								
27	***a*·*a b*·*b c*·*c***	***b*·*c***	$\frac{a\cdot a}{2}$	$\frac{a\cdot a}{2}$	+	Monoclinic	*I*^f^	$0\bar{1}1/\bar{1}00/1\bar{1}\bar{1}$
28	***a*·*a b*·*b c*·*c***	$\frac{a\cdot b}{2}$	$\frac{a\cdot a}{2}$	***a*·*b***	+	Monoclinic	*C*	$\bar{1}00/\bar{1}02/010$
29	***a*·*a b*·*b c*·*c***	$\frac{a\cdot c}{2}$	***a*·*c***	$\frac{a\cdot a}{2}$	+	Monoclinic	*C*	$100/1\bar{2}0/00\bar{1}$
30	***a*·*a b*·*b c*·*c***	$\frac{b\cdot b}{2}$	$\frac{a\cdot b}{2}$	***a*·*b***	+	Monoclinic	*C*	$010/01\bar{2}/\bar{1}00$
37	***a*·*a b*·*b c*·*c***	−|***b*·*c***|	$-\frac{a\cdot a}{2}$	0	−	Monoclinic	*C*^c^	102/100/010
39	***a*·*a b*·*b c*·*c***	−|***b*·*c***|	0	$-\frac{a\cdot a}{2}$	−	Monoclinic	*C*^d^	$\bar{1}\bar{2}0/\bar{1}00/00\bar{1}$
41	***a*·*a b*·*b c*·*c***	$-\frac{b\cdot b}{2}$	−|***a*·*c***|	0	−	Monoclinic	*C*^b^	$0\bar{1}\bar{2}/0\bar{1}0/\bar{1}00$
43	***a*·*a b*·*b c*·*c***	$-\frac{b\cdot b-\left|a\cdot b\right|}{2}$	$-\frac{a\cdot a-\left|a\cdot b\right|}{2}$	−|***a*·*b***|	−	Monoclinic	*I*	$\bar{1}00/\bar{1}\bar{1}\bar{2}/0\bar{1}0$

a Derived from the metric classification of the 44 reduced forms [1,2].

$\begin{array}{c} {\;}^{b}\text{If}\;a\cdot a<4\left|a\cdot c\right|\\ {\;}^{c}\text{If}\;b\cdot b<4\left|b\cdot c\right|\\ {\;}^{d}\text{If}\;c\cdot c<4\left|b\cdot c\right| \end{array}\}$ Premultiply table matrix by $00\bar{1}/010/101$ (*I*-centered).

$\begin{array}{c} {\;}^{e}\text{If}\;3a\cdot a<c\cdot c+2\left|a\cdot c\right|\\ {\;}^{f}\text{If}\;3b\cdot b<c\cdot c+2\left|b\cdot c\right| \end{array}\}$ Premultiply table matrix by $\bar{1}0\bar{1}/010/100$ (*C*-centered).

g No required relationships between symmetrical scalars for reduced forms 27–44.

h For the conventional cell in the monoclinic system, *b* is taken as the *unique* axis, and *a* and *c* are chosen coincident with the shortest two translations in the net perpendicular to *b*. The angle *β* is taken to be non-acute. This choice allows primitive, side-centered, and body-centered lattices. In the primitive and body-centered lattices *a* and *c* obey *a* < *c*. The side-centered lattice is taken as *C*-centered.

**Table 2:** Crystallographic parameters reported for five centered monoclinic cells selected from the recent literature [8–12]. The Table shows that each literature *C*-centered cell can be transformed to a conventional *I*-centered cell in which the *β* angle is closer to 90°. Numbers in parentheses represent standard deviations

No.	1[8]	2[9]	3[10]	4[11]	5[12]

	Lattice I	Lattice II	Lattice III	Lattice IV	Lattice V
Literature cells−monoclinic *C*-centered					

Cell	LC1	LC2	LC3	LC4	LC5
*a*(Å)	21.4179(6)	19.3044(4)	14.744(3)	27.4540(10)	23.785(9)
*b*(Å)	11.1466(2)	10.6009(3)	8.850(3)	12.2738(10)	13.610(3)
*c*(Å)	14.9649(3)	11.6549(3)	10.062(3)	16.2792(10)	19.080(7)
*β*(°)	133.4280(6)	125.527(2)	131.609(19)	123.118(10)	121.927(12)
*V*(Å^3^)	2594.61(10)	1941.10(8)	981.7(5)	4594.4(5)	5242(3)
Sp. Gr.	*C*2	*Cc*	*C*2/*c*	*C*2/*c*	*C*2
Yr. Pub.	2001	2000	2000	2001	2001

Standard conventional cells−monoclinic *I*-centered					

Cell	SC1	SC2	SC3	SC4	SC5
*a*(Å)	14.965	11.655	10.062	16.279	19.08
*b*(Å)	11.147	10.601	8.850	12.274	13.610
*c*(Å)	15.556	15.717	11.027	23.029	21.208
*β*(°)	90.89	91.59	91.41	93.18	107.85
*V*(Å^3^)	2594.61	1941.10	981.7	4594.4	5242
Sp. Gr.	*I*2	*Ia*	*I*2/*a*	*I* 2/*a*	*I*2

Reduced forms^a^					

Form	RF1	RF2	RF3	RF4	RF5
***a*·*a***	1.00	1.00	1.00	1.00	1.00
***b*·*b***	1.17	1.08	1.00	1.50	1.01
***c*·*c***	1.20	1.12	1.04	1.64	1.68
***b*·*c***	0.29	0.50	−0.20	0.69	0.37
***a*·*c***	0.50	0.50	−0.33	0.50	0.50
***a*·*b***	0.50	0.50	−0.47	0.50	0.50
Form No.	27	27	17	27	27

a Reduced forms have been normalized.

## Footnotes

1 In certain cases, there are trivial deviations from strict adherence to the conventions such as relabeling of the axes (e.g. the interchange of the *a* and *c*-axes so as to use *P*2_1_/*c* rather than *P*2_1_/*a*).
